# Multitarget Mechanisms of (‒)‒Epigallocatechin-3-Gallate Against MRSA: From SraP L-Lectin Targeting to Synergistic Antibiotic Effects

**DOI:** 10.3390/pathogens15010090

**Published:** 2026-01-13

**Authors:** Ping Zheng, Peihua Zhang, Yuan Li, Jinzhao Long, Fang Liu, Haiyan Yang

**Affiliations:** Department of Epidemiology, School of Public Health, Zhengzhou University, Zhengzhou 450001, China

**Keywords:** L-Lectin, MRSA, (‒)‒epigallocatechin-3-gallate, SraP, virulence

## Abstract

Methicillin-resistant *Staphylococcus aureus* (MRSA), a major global public health threat due to its broad resistance, urgently requires the development of new antibiotic alternatives. (‒)‒Epigallocatechin-3-gallate (EGCG) is considered a natural bioactive compound with anti-MRSA properties. The L-Lectin module of serine-rich adhesin for platelets (SraP) is considered an important target for blocking MRSA-infected hosts. This study aims to investigate the mechanism of action of EGCG against MRSA. Surface plasmon resonance (SPR), cell adhesion and invasion, biofilm formation, checkerboard assays, RNA sequencing (RNA-seq) and quantitative real-time polymerase chain reaction (qRT-PCR) were performed. The results showed that EGCG bound to SraP L Lectin with high affinity and effectively inhibited MRSA colonization. Additionally, EGCG significantly suppressed pyrimidine metabolism and downregulated related genes, thereby potentially inhibiting bacterial growth. It also markedly reduced the expression of multiple genes associated with β-lactam resistance and inhibited biofilm formation. A strong synergistic effect was observed between EGCG and the bactericidal agent ceftriaxone (CRO). When combined with 10 μg/mL EGCG, CRO required 75% less dosage and exhibited a prolonged antimicrobial effect. In conclusion, EGCG exerts anti-MRSA effects through multiple pathways and represents a promising candidate as an alternative therapeutic agent against MRSA infections.

## 1. Introduction

*Staphylococcus aureus* (*S. aureus*) is a bacterium widely present in humans, animals, and the environment, which exists not only as a commensal organism but also as a pathogen capable of causing infections ranging from localized purulent conditions to severe systemic diseases such as sepsis [1]. The widespread use of antibiotics such as penicillin has led to the rapid evolution of *S. aureus*, and methicillin-resistant *S. aureus* (MRSA) with a wide resistance spectrum and high resistance rate has emerged [2]. Currently, MRSA causes approximately 15% of hospital-acquired invasive infections worldwide and is closely associated with high morbidity and mortality [3,4]. It is noteworthy that MRSA is resistant not only to beta-lactams but also to other classes like aminoglycosides and fluoroquinolones in many strains, due to the acquisition of specific resistance genes [5,6]. This significantly limits treatment options and leads to a higher disease burden. Currently, antibiotics remain the primary means of combating MRSA infections, but overuse and improper use accelerate the evolution and spread of antibiotic-resistant bacteria [7,8]. Of greater concern, though relatively rare, is the emergence of vancomycin-resistant *S. aureus*, which poses a severe threat by further narrowing the treatment options when it occurs [9]. Antibiotic resistance has become a global public health crisis. The continuous emergence of new resistance mechanisms and the scarcity of effective antibacterial drugs severely threaten the treatment capabilities for common infections as well as major diseases [10]. Thus, the urgent need for developing novel antibiotic alternatives and discovering new antimicrobials to combat resistant pathogens is clear.

Virulence factors play an important role in the pathogenesis of *S. aureus* [11,12]. In recent years, anti-virulence therapy has garnered widespread attention from all sectors of society. This therapeutic approach reduces the pathogenicity of pathogens, thereby minimizing their damage to the host organism without exerting survival pressure on the pathogenic bacteria, making it less likely for resistance to develop. It offers a new perspective for reducing bacterial resistance to conventional antibiotics and holds promise as a novel strategy for treating *S. aureus* infections [13,14,15]. Given the potential of biologically active natural compounds from plants in anti-MRSA research, targeting key virulence factors has become an important direction for novel antibacterial strategies [16,17]. Virulence factors include cell wall-anchoring proteins (CWAs) involved in bacterial adhesion, extracellular toxins and enzymes that promote tissue necrosis, and other bacterial structures such as capsular polysaccharides, biofilms, etc., that interfere with host immune responses [18,19], among which CWA is an important virulence factor for *S. aureus* to survive in a symbiotic state and during aggressive infection, providing an important opportunity for bacteria-host interactions, and targeting CWA can fight *S. aureus* infection [18]. Serine-rich adhesin for platelets (SraP) is a CWA that mediates the adhesion of bacteria to host cells, affects the formation of bacterial biofilm, interferes with host innate and adaptive immune responses, promotes the inflammatory response of the host body, and plays an important role in the pathogenesis of bacteria [20,21]. The L-Lectin module located in the SraP protein ligand binding region mediates the binding of *S. aureus* to host cells through the specific binding of key amino acids such as Try367 to the monosaccharide N-acetylneuraminic acid (Neu5Ac) on the surface of the cell membrane. Silencing their genes can inhibit or eliminate bacterial infection of host cells. Anti-SraP L-Lectin antibodies against the L-Lectin module are effective in blocking MRSA invasion and infection of the host [21,22]. Therefore, in-depth exploration of the regulatory effect of natural bioactive compounds on virulence factors such as L-Lectin will provide an important theoretical basis for the development of novel anti-MRSA drugs based on biologically active natural compounds from plants.

Plant natural bioactive compounds have gradually become the focus of public health due to their unique antimicrobial mechanisms and low resistance induction potential [23,24]. Numerous studies have shown that many plant extracts and their biologically active natural compounds, such as flavonoids, alkaloids, and polyphenols, not only directly inhibit MRSA growth, but disrupt biofilm formation, enhance host immune responses, and even reverse bacterial resistance [25,26,27]. Therefore, it is highly significant to systematically study the anti-MRSA mechanism of plants’ natural bioactive compounds and evaluate their application potential in public health practice to curb the spread of drug-resistant bacteria and reduce the burden of infection. (‒)‒Epigallocatechin-3-gallate (EGCG) is a major bioactive polyphenol most abundant in green tea, and it has also been identified in other plant sources such as grapes [28,29]. Studies have shown that EGCG has a significant antibacterial effect on *S. aureus* [28,30,31,32,33,34]. EGCG weakened the hemolytic effect of Hla and inhibited *S. aureus* by suppressing the secretion of Hla, and significantly reduced the excessive production of reactive oxygen species (ROS) and the activation of the MAPK (mitogen-activated protein kinase) signaling pathway induced by Hla, thereby significantly reducing the expression of NLRP3 (NOD-, LRR- and pyrin domain-containing protein 3) inflammasome-related proteins in THP-1 cells [28]. However, studies targeting the SraP L-Lectin domain of MRSA with EGCG are scarce.

Thus, this study aimed to elucidate the molecular mechanisms underlying EGCG’s anti-MRSA activity, with a particular focus on characterizing its interaction with the SraP L-Lectin domain. Additionally, we sought to investigate other putative pathways through which EGCG exerts its antibacterial effects.

## 2. Materials and Methods

### 2.1. Bacterial Strains and Culture Conditions

The MW2 strain was sourced from Biosea (Beijing, China). Eleven clinical MRSA isolates used in this study were preserved in the laboratory of the School of Public Health, Zhengzhou University. The minimum inhibitory concentrations (MICs) of these 12 strains against different antimicrobial agents are listed in Table S1. Frozen stocks were streaked onto Mueller-Hinton agar (MHA) plates. A single colony was inoculated into Mueller-Hinton broth (MHB) and cultured at 37 °C with shaking at 200 rpm for 3 h. The resulting bacterial suspension was used for subsequent experiments.

A549 cells were cultured in Dulbecco’s modified Eagle’s medium (DMEM, Gibco, Waltham, MA, USA) supplemented with 10% fetal bovine serum (FBS), 5 mM glutamine, 5 µg/mL penicillin, and 100 µg/mL streptomycin. These cells were used for adhesion and invasion assays.

### 2.2. SraP L-Lectin Domain Analysis

SraP L-lectin modules were amplified from all 12 MRSA strains, using the primer pair, GTGCCGCAGTAGGTATTGG (F) and ACGTTGTCGCAACACCATAA (R). The PCR products were purified and sequenced using an ABI 3700 sequencer. The obtained DNA sequences were translated into their corresponding amino acid sequences. These derived amino acid sequences were subsequently compared using MEGA v6.0, with the final comparative results generated via Clustal X (v2.1).

### 2.3. Construction of ΔL-Lectin Mutant

The amplicons flanking the Srap L-Lectin locus were cloned into the *S. aureus* pKOR1 vector as previously published [35]. Briefly, the upstream and downstream flanking regions of the SraP L-Lectin gene were amplified by polymerase chain reaction (PCR) using specific primer pairs (L-Lectin-up-F/R and L-Lectin-down-F/R; see Table S2). The PCR products were purified, ligated together using overlap extension PCR, and subsequently cloned into the temperature-sensitive *S. aureus* vector pKOR1. The resulting plasmid, pKOR1-ΔL-Lectin, was first transformed into *Escherichia coli* (*E. coli*) DH5α for propagation and then electroporated into *S. aureus* MW2. Positive clones were selected at 30 °C on tryptic soy agar (TSA) plates containing 10 μg/mL chloramphenicol. Allelic exchange was induced as follows: a single colony was inoculated into tryptic soy broth (TSB) with chloramphenicol and grown overnight at 42 °C (the non-permissive temperature) to facilitate plasmid integration into the chromosome. The culture was then diluted and plated on TSA plates containing 100 ng/mL anhydrotetracycline at 37 °C to induce the second crossover event and counterselect against the plasmid. Potential knockout mutants were screened by colony PCR. The complete deletion of the SraP L-Lectin gene was finally confirmed by DNA sequencing and quantitative real-time PCR (qRT-PCR).

### 2.4. Surface Plasmon Resonance (SPR) Assays

SPR was conducted on a Biacore T200 instrument (GE Healthcare, Chicago, IL, USA) at 25 °C to evaluate the binding affinities between Srap L-Lectin and EGCG. The following buffers were purchased from Cytiva (Marlborough, MA, USA): Concentrated 10x HBS-P+ stock (0.1 M 4-(2-Hydroxyethyl)piperazine-1-ethanesulfonic acid, 1.5 M NaCl, 0.5% *v*/*v* surfactant Tween 20, pH 7.4); screening buffer (1x HBS-P+, pH 7.4, with 5% dimethyl sulfoxide (DMSO)); and regeneration solution (10 mM glycine-HCl, pH 3.0). Srap L-Lectin was immobilized covalently on a CM5 sensor chip via amine coupling kit. The chip was activated with the N-ethyl-N′-[3-dimethylaminopropyl] carbodi-imide/N-hydroxysuccinimide solution. Srap L-Lectin was injected at a flow rate of 5 µL/min, diluted in 10 mM acetate buffer (pH 5.5). A reference flow cell was subjected to the same procedure without protein to serve as a blank. After immobilization, the surface was blocked with 1 M ethanolamine-HCl. EGCG was diluted in running buffer and injected after a 1 min incubation period. Both association and dissociation phases were monitored for 300 s each. The sensor surface was regenerated with 50 mM NaOH. Neu5Ac, a known natural ligand of Srap L-Lectin, was used as a positive control. Sensorgram data were processed and fitted globally using the Biacore T200 Evaluation Software (v4.1). The association rate (*k_a_*) and dissociation rate (*k_d_*) were calculated by the BiaEvaluate software (v4.1) according to the kinetic parameters and resultant sensorgrams. The equilibrium dissociation constant (Kd) was calculated as the ratio *k_d_/k_a_*.

### 2.5. CFU-Based Bacterial Growth Assay

MRSA suspensions were standardized to 2 × 10^6^ CFU/mL in MHB medium, with concentration verified by plate counting. For single-agent evaluation, filter-sterilized EGCG (1000 μg/mL stock) was diluted to final concentrations of 250, 125, 50, 10, 5, and 2 μg/mL, while control groups received MHB only. Combination studies tested: (1) control, (2) 10 μg/mL EGCG, (3) 10 μg/mL ceftriaxone, (4) 20 μg/mL ceftriaxone, and (5–8) EGCG (10 μg/mL) with ceftriaxone (3–10 μg/mL). All groups maintained 1 × 10^6^ CFU/mL initial inoculum in 37 °C shaking cultures (200 rpm). Growth kinetics were monitored at 0, 2, 4, 6, and 8 h through serial PBS dilution plating and 37 °C overnight incubation for CFU enumeration.

### 2.6. Adhesion and Invasion Assays

**Figure 1:**
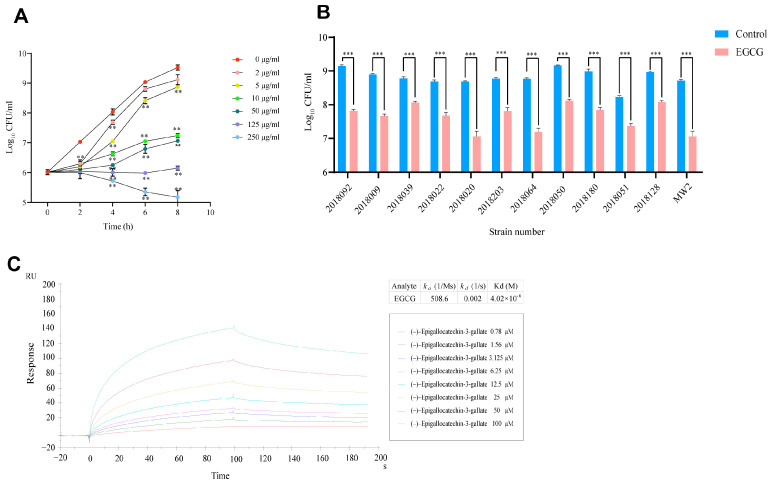
Inhibitory effect of EGCG on MRSA MW2 growth and its interaction with the target protein L-Lectin. (**A**) CFU-based bacterial growth curve of wild-type (WT) MRSA strain MW2 under different concentrations of EGCG inhibition. (**B**) The growth inhibitory effect of 10 µg/mL EGCG on clinical isolates of MRSA. (**C**) Surface plasmon resonance (SPR) sensorgram for kinetic analysis of EGCG binding to L-Lectin of MW2. Control: PBS-treated MRSA strains. EGCG: 10 μg/mL EGCG-treated MRSA strains. CFU: colony-forming units. RU: response or resonance unit. ** *p* < 0.01, *** *p* < 0.001. The number represents the mean ± SD of four replicates.

Adhesion and invasion experiments were performed as described previously [21]. The concentration of 10 μg/mL EGCG was selected for these assays based on our preliminary growth kinetics data, which identified it as a sub-inhibitory concentration that potently suppressed MRSA growth (Figure 1A).

(1) Adhesion Assay: A549 monolayers with 2.5 × 10^5^ cells per well were seeded in a 24-well plate. The medium was aspirated, cells were washed three times with PBS (phosphate-buffered saline), and bacterial suspensions were added (either control or 10 μg/mL EGCG-treated *S. aureus* at 6.25 × 10^8^ CFU/mL, diluted 1:100 in DMEM with 10% FBS). Cells were incubated at 37 °C with 5% CO_2_ for 2 h. DMEM was aspirated, and cells were washed five times with PBS and then digested with 200 μL of trypsin (2.5 mg/mL) for 2 min. Cells were resuspended in 800 μL of PBS, diluted and plated. Colonies were counted after overnight incubation.

(2) Invasion Assay: A549 monolayers were prepared in a 96-well plate. Following washing, bacterial suspensions were added (either control or EGCG-treated) and cells were incubated at 37 °C with 5% CO_2_ for 2 h. Then, gentamicin at 100 μg/mL was added and maintained for 2 h to eliminate extracellular bacteria. Cells were washed five times with PBS, treated with 200 μL of trypsin (2.5 mg/mL) for 2 min, resuspended in 800 μL of PBS, diluted, and plated. Colonies were counted after overnight incubation.

### 2.7. Minimum Inhibitory Concentration (MIC) Assay

The MICs of compounds were determined using a microdilution method as well as agar plating [36]. 100 μL of bacterial strains in MHB at a concentration of 5 × 105 CFU/mL was added to 96-well plates. Subsequently, 100 μL of culture media containing various concentrations of test compounds and reference antibiotics was added to the respective wells. The MIC in this study is defined as the lowest concentration that confers a complete inhibition of colony growth on agar plates (i.e., 100% reduction in colony counts in all replicates or MIC100).

### 2.8. Biofilm Formation Assay

The concentrations of WT and ΔL-Lectin strains were adjusted to 2 × 10^6^ CFU/mL in TSB medium containing 0.5% sucrose (verified by plating). Subsequently, medium with different concentrations of EGCG (500, 250, 100, 20, 10, 4, 0 µg/mL) was added to achieve a final bacterial concentration of 1 × 10^6^ CFU/mL. Different concentrations of bacterial suspensions were added to 96-well plates as experimental groups and corresponding sterile media as negative control groups. Plates were incubated at 37 °C for 24 h and the biofilm formation index (BFI) was determined: bacteria were washed with PBS, stained with 0.1% crystal violet for 20 min, and decolorized with ethanol. The OD_570_ was measured. BFI was calculated using the formula BFI = (S−SC)/(G−GC) (where S/G represents the staining/culture OD values of the experimental group, and SC/GC represents the OD values of the negative control group). A BFI > 1.10 indicates strong biofilm, 0.70~1.09 indicates moderate, 0.35~0.69 indicates weak, and <0.35 indicates none [37]. Further, the inhibitory effect of EGCG on biofilm formation by the MW2 strain at different exposure times and clinical strains using the lowest compound concentration that achieves ≥90% inhibition was tested.

### 2.9. Extraction of Total RNA from S. aureus

Total RNA from biofilm-forming and planktonic bacteria was extracted using the Trizol method. First, 200 μL of lysozyme (100 mg/μL) was added to bacterial pellets to disrupt the cell wall, followed by incubation at 37 °C for 2 h. Then, 1 mL of Trizol and grinding beads were added, and the mixture was ground in a grinder at 60 Hz for 60 s, followed by chilling on ice for 10 min. Subsequently, 300 μL of chloroform was added, and the mixture was centrifuged at 12,000× *g* at 4 °C for 15 min. In addition, 500 μL of colorless aqueous phase was transferred into the tubes, 500 μL of isopropyl alcohol was added, and centrifugation was performed at 12,000× *g* for 15 min at 4 °C. After discarding the supernatant, the RNA pellet was washed with 1 mL of 75% ethanol and centrifuged at 10,000× *g* for 5 min at 4 °C. The Spin Column RNA Cleanup & Concentration Kit (Sangon, Shanghai, China) was used to purify the RNA. Extracted RNA concentration and quality were determined using a Nanodrop 2000 spectrophotometer (Thermo Fisher Scientific, Waltham, MA, USA). The RNA quality met the requirements, with an A260/A280 ratio of about 2.0. Finally, RNA reverse transcription was performed by following the operating instructions for the Hifair^®^ III 1st Strand cDNA Synthesis Super Mix for qPCR (gDNA digester plus) kit.

### 2.10. RNA Sequencing and Bioinformatics

Strains were cultured in TSB medium to OD_600_ of 0.5–0.8 at a temperature of 37 °C and a rotational speed of 180 rpm. After 4–6 h, 2 mL of bacteria were collected for RNA sequencing with 5 parallel samples per strain. The RNA extracted in 2.3 was sequenced on the Illumina NovaSeq 6000 platform. DESeq2 (1.20.0) software was used to analyze the differential expression between the control group and the EGCG group. The *p* value (Padj) was adjusted using the Benjamini and Hochberg methods to control the false detection rate. Padj < 0.05 or |log2(foldchange)| > 1 was set as the threshold for significant differential expression. The clusterProfiler (3.8.1) software was used to analyze the statistical enrichment of differentially expressed genes in the KEGG pathway.

### 2.11. qRT-PCR Assay

The relative expression levels of genes were measured using qRT-PCR, following the manufacturer’s guidelines for ChamQ Universal SYBR qPCR Master Mix. The reaction protocol included an initial denaturation at 95 °C for 30 s, followed by 40 cycles of 10 s at 95 °C and 30 s at 60 °C. The relative expression of the genes was calculated using the 2^−ΔΔCT^ method [38], with 16S rRNA serving as the internal control. All reactions were performed in triplicate. The primer sequences utilized in the qRT-PCR experiments are detailed in Table S3.

### 2.12. Checkerboard Assay

The synergistic interaction between antibiotics and natural bioactive compounds was assessed using a standardized checkerboard microdilution method. Bacterial suspensions were adjusted to 2 × 10^6^ CFU/mL in MHB medium. In 96-well microplates, antibiotics were diluted vertically (columns 1–12) in two-fold serial dilutions ranging from 1/2 MIC to 1/64 MIC, while natural compounds were diluted horizontally (rows A–H) from 1024 μg/mL to 4 μg/mL. Each well, except sterile controls (row A) and growth controls (column 12), was inoculated with 100 μL bacterial suspension to achieve a final concentration of 1 × 10^6^ CFU/mL. Following 24 h incubation at 35 °C, bacterial growth was determined by measuring OD_600_. Synergistic effects were quantified using the fractional inhibitory concentration index (FICI). The calculation is as follows:

$${FIC}_{EGCG}=\frac{{MIC}_{combine}}{{MIC}_{EGCG}}$$

$${FIC}_{antibiotic}=\frac{{MIC}_{combine}}{{MIC}_{antibiotic}}$$

$$FICI={FIC}_{EGCG}+{FIC}_{antibiotic}$$

where FIC_EGCG_ and FIC_antibiotic,_ respectively, represent the FIC of the natural active compound EGCG and antibiotics. MIC_combine_ represents the MIC of EGCG and antibiotic combination. FICI is commonly used to define drug synergistic effects that inhibit bacteria. Interactions were classified as: synergistic (FICI ≤ 0.5), additive (0.5 < FICI ≤ 1), indifferent (1 < FICI ≤ 2), or antagonistic (FICI > 2) [39].

### 2.13. Statistical Analysis

Data statistical analysis was performed using GraphPad Prism 8.3.0 software. Quantitative data are expressed as mean ± standard deviation (SD). Two-group comparisons were conducted using *t*-tests, while multiple groups compared to a control group were analyzed using one-way ANOVA. The bacterial proliferation rate was compared using two-way ANOVA. The significance level α was set at 0.05, with differences considered statistically significant at *p* ≤ 0.05.

## 3. Results

### 3.1. EGCG Inhibited MRSA Growth Independent of L-Lectin Binding

We first tested the inhibitory effect of EGCG on the growth of MRSA. The results showed that EGCG directly suppressed bacterial growth in a dose-dependent manner (Figure 1A). At a concentration of 10 µg/mL, which was selected for further analysis, EGCG treatment strongly inhibited the growth of MW2. The viable bacterial counts at 4, 6, and 8 h were 1.44, 1.98, and 2.28 log_10_ lower than those of the control, respectively. The activity spectrum of EGCG at a concentration of 10 µg/mL was assessed by testing against MW2 and 11 clinical MRSA isolates. EGCG treatment achieved growth inhibition rates ranging from 80.87% to 97.69% across different strains (Figure 1B).

We next investigated the binding characteristics between EGCG and the Srap L-Lectin protein using SPR technology. As shown in Figure 1C, the equilibrium dissociation constant (Kd) for the binding of EGCG to the protein was determined to be 4 × 10^−6^ M. This value is lower than the dissociation constant reported in the literature for the natural ligand Neu5Ac binding to L-Lectin (5.4 × 10^−4^ M) [21], suggesting that EGCG may exhibit stronger binding affinity for L-Lectin compared to its natural ligand. To further investigate the potential role of the L-Lectin pathway, we compared the efficacy of EGCG against the WT and ΔL-Lectin strains. Although a trend of slightly attenuated inhibition was observed in the ΔL-Lectin strains (92.10%) relative to the WT controls (92.69%), this apparent difference did not reach significance (*p* = 0.292) (Figure S1). This result implied EGCG may have other mechanisms for inhibiting the growth of MRSA that are independent of the L-Lectin module.

### 3.2. EGCG Inhibited MRSA Adhesion to and Invasion of A549 Cells

**Figure 2:**
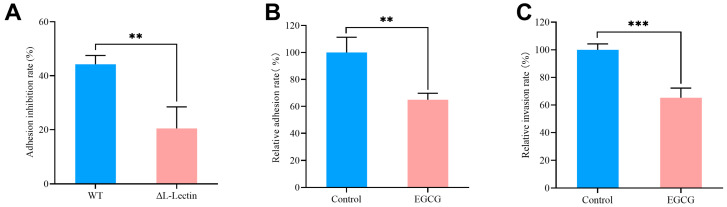
Effects of EGCG on the adhesion and invasion of MRSA to A549 cells (**A**) Comparison of adhesion inhibition rates between the WT group and the ΔL-Lectin group. (**B**) Comparison of relative adhesion rates between the control group and the EGCG group. (**C**) Comparison of relative invasion rates between the control group and the EGCG group. WT: 10 μg/mL EGCG-treated MW2 strains. ΔL-Lectin: 10 μg/mL EGCG-treated ΔL-Lectin strains. Control: PBS-treated MRSA strains. EGCG: 10 μg/mL EGCG-treated MRSA strains. ** *p* < 0.01, *** *p* < 0.001. The number represents the mean ± SD of four replicates.

Based on the binding characteristics of EGCG and L-Lectin protein, we speculated that its function might be more likely to interfere with the bacterial adhesion process. Therefore, we investigated the effect of EGCG on bacterial adhesion and invasion capabilities. Comparative infection assays using MRSA MW2 WT strains and ΔL-Lectin strains, both pretreated with EGCG, showed that the inhibitory effect on adhesion was significantly weaker in the ΔL-Lectin strains (*p* = 0.0211), indicating the anti-adhesive effect of EGCG depended on the presence of the L-Lectin protein. (Figure 2A). In addition, EGCG treatment significantly reduced MW2 adhesion by 34% (*p* = 0.0023) and invasion by 35% (*p* = 0.0007) compared to PBS-treated controls (Figure 2B,C).

### 3.3. EGCG Inhibited MRSA Biofilm Formation

**Figure 3:**
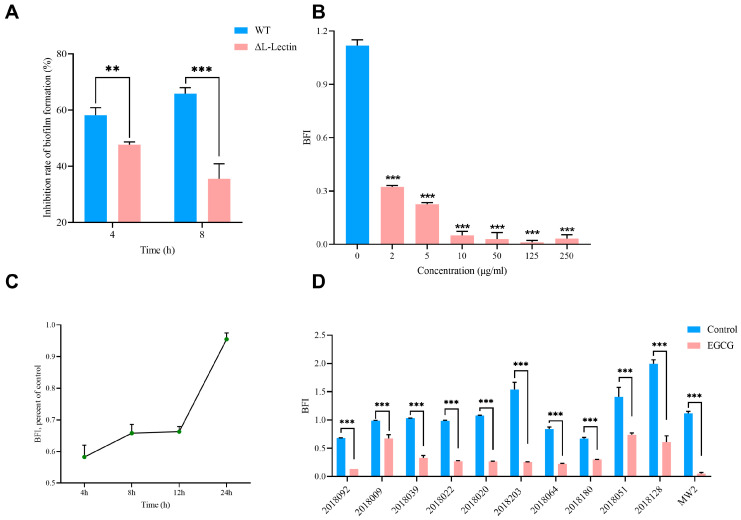
The inhibitory effect of ECGC on MRSA biofilm formation. (**A**) The comparison of biofilm formation inhibition rates between the WT group and the ΔL-Lectin group was greater at 8 h observed with 10 μg/mL EGCG intervention than that at 4 h. (**B**) The inhibitory effect of different concentrations of ECGC on MW2 biofilm formation. (**C**) The inhibitory effect of 10 µg/mL ECGC on MW2 biofilm formation at different time points. (**D**) The effect of 10 µg/mL EGCG on biofilm formation by different MRSA strains after 24 h. BFI: biofilm formation index. Control: PBS-treated MRSA strains. EGCG: 10 μg/mL EGCG-treated MRSA strains. WT: 10 μg/mL EGCG-treated MW2 strains. ΔL-Lectin: 10 μg/mL EGCG-treated ΔL-Lectin strains. ** *p* < 0.01, *** *p* < 0.001. The number represents the mean ± SD of four replicates.

Biofilm formation is a critical step in MRSA pathogenesis. To investigate whether EGCG affects MRSA biofilms through the L-Lectin module, we compared biofilm inhibition between WT and ΔL-Lectin strains after 4 h and 8 h of treatment with 10 µg/mL EGCG. The results showed EGCG treatment inhibited biofilm formation in both WT and ΔL-Lectin strains, and the inhibitory effect of EGCG on biofilm formation was significantly greater in the WT strain than in the ΔL-Lectin mutant, with a difference of 10.49% at 4 h (*p* = 0.007) and 30.32% at 8 h (*p* < 0.0001) (Figure 3A). We further assessed the changes in BFI after treating the strains with EGCG. Figure 3B showed that MW2 is a robust biofilm-forming strain, with a BFI exceeding 1.1. Notably, treatment with 2 µg/mL EGCG for 24 h reduced the BFI to 0.324, representing 71.34% inhibition (*p* < 0.0001). At concentrations of 10 µg/mL or higher, EGCG inhibited biofilm formation by more than 90% (*p* < 0.0001) (Figure 3B). In addition, extended exposure to EGCG resulted in progressively stronger suppression of biofilm formation (Figure 3C). A 10 µg/mL concentration of EGCG inhibited biofilm formation not only in the MW2 strain but also in clinical MRSA isolates (Figure 3D). The tested isolates comprised three distinct biofilm-forming phenotypes: two weak formers (0.35 ≤ BFI < 0.70), five moderate formers (0.7 ≤ BFI < 1.10), and three strong formers (BFI ≥ 1.10) [37]. Furthermore, EGCG treatment converted 80% of strains to non-biofilm formers (BFI < 0.35). Notably, one moderate biofilm former (MRSA 2018009) and one strong biofilm former (MRSA 2018128) were downgraded to weak formers, while another strong biofilm former (MRSA 2018051) became a moderate former. The results demonstrated that beyond quantitatively inhibiting biofilm formation, EGCG also qualitatively impaired the biofilm properties of clinical MRSA strains.

### 3.4. EGCG Exerted Its Anti-MRSA Effect by Suppressing Pyrimidine Metabolism, Virulence Factors, and Biofilm-Related Genes

**Figure 4:**
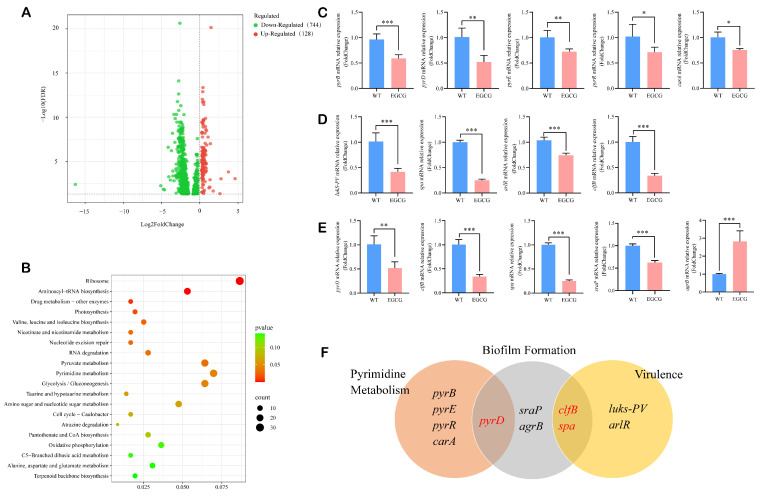
RNA-seq and qRT-PCR analyses revealed the multifaceted mechanisms of EGCG-mediated inhibition of MRSA. (**A**) Volcano plot of all differentially expressed genes. (**B**) KEGG pathway enrichment analysis of DEGs. (**C**) Expression levels of pyrimidine metabolism related genes measured by qRT-PCR. (**D**) Expression levels of virulence genes measured by qRT-PCR. (**E**) Expression levels of biofilm formation related genes measured by qRT-PCR. (**F**) Venn diagram of key genes involved in pyrimidine metabolism, virulence, and biofilm formation. WT: PBS-treated MW2 strains. EGCG: 10 μg/mL EGCG-treated MW2 strains. * *p* < 0.05, ** *p* < 0.01, *** *p* < 0.001. The number represents the mean relative expression (or fold changes ± SD) of four replicates.

To move beyond phenotypic observations and gain mechanistic insights into the action of EGCG, we performed transcriptome sequencing on EGCG-treated and untreated WT strains. EGCG treatment induced transcriptional alterations in 872 genes (744 downregulated and 128 upregulated; Figure 4A). Among these, 358 differentially expressed genes (DEGs) were mapped to 114 KEGG pathways. Fifteen pathways were identified as significantly enriched, including the ribosome, drug metabolism–other enzymes, pyrimidine metabolism, and glycolysis/gluconeogenesis pathways (*p* < 0.05; Figure 4B).

De novo nucleotide biosynthesis is critical for bacterial virulence and represents a potential anti-virulence target [40]. We found the genes associated with de novo pyrimidine nucleotide synthesis showed pronounced suppression including *pyrB*, *pyrD*, *pyrE*, *pyrR*, and *carA*, a finding corroborated by qRT-PCR validation (Figure 4C and Figure S2A). Therefore, EGCG may inhibit MRSA growth and proliferation by suppressing de novo pyrimidine nucleotide synthesis, thus reducing the availability of essential precursors and affecting DNA replication and transcription processes. Moreover, EGCG also markedly altered virulence genes in MW2. Key genes encoding adhesion-promoting CWAs, including *spa* and *clfB*, were significantly downregulated (Figure 4D and Figure S2B). Significant suppression was also observed for *arlR*, a component of a two-component regulatory system, and *lukS-PV*, which encodes a leukocidin subunit. Besides virulence genes, we further analyzed genes associated with biofilm formation. The expression of several key biofilm-related genes was also significantly downregulated following EGCG treatment, such as *pyrD*, *clfB*, and *spa*. A discrepancy was observed between the transcriptomic and qRT-PCR results for *sraP*. Despite the lack of a significant change in the RNA-seq dataset, the downregulation of *sraP* was confirmed to be significant by qRT-PCR (Figure 4E and Figure S2C). Notably, some genes involved in both pyrimidine metabolism and virulence are also implicated in biofilm formation, such as *pyrD*, *clfB*, and *spa* [41,42] (Figure 4F). Furthermore, the expression of the quorum sensing gene *agrB* is significantly upregulated (Figure 4E and Figure S2C). Notably, for all genes validated by qRT-PCR, the directional trend of expression change (up- or down-regulation) was fully concordant between the two methodologies, despite variations in the absolute fold-change magnitudes. Taken together, our results demonstrated that EGCG combated MRSA by disrupting the pyrimidine metabolism pathway while concurrently inhibiting the virulence and biofilm formation.

### 3.5. EGCG Suppressed Resistance Genes and Synergized with Ceftriaxone to Combat MRSA

**Figure 5:**
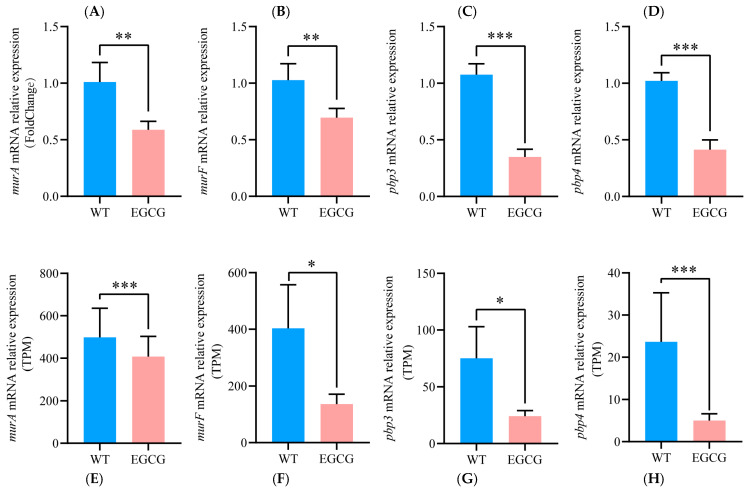
The expression levels of genes related to resistance against beta-lactam antibiotics after EGCG intervention. (**A**–**D**) represent the genes expression levels related to β-lactam antibiotic resistance detected by qRT-PCR; (**E**–**H**) show the comparison of transcriptomic detection results for corresponding genes. TPM: transcripts per million. WT: PBS-treated MW2 strains. EGCG: 10 μg/mL EGCG-treated MW2 strains. * *p* < 0.05, ** *p* < 0.01, *** *p* < 0.001. The number represents the mean relative expression (or fold changes ± SD) of four replicates.

**Figure 6:**
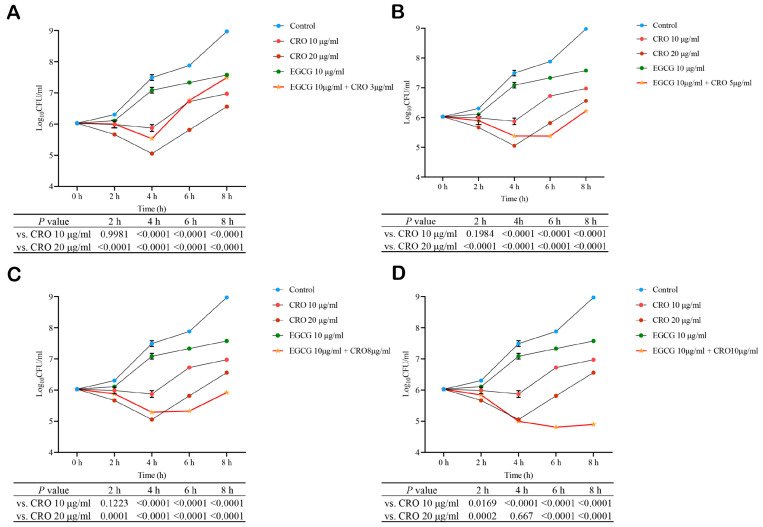
CFU-based bacterial growth curve of wild-type (WT) MRSA strain MW2 under treatment with EGCG, ceftriaxone, and their combinations. Each panel compares the growth kinetics of control (MHB only), 10 µg/mL ceftriaxone (10 CRO), 20 µg/mL ceftriaxone (20 CRO), 10 µg/mL EGCG (10 EGCG), and a fixed concentration of EGCG (10 µg/mL) in combination with increasing concentrations of CRO: (**A**): 10 EGCG + 3 µg/mL CRO. (**B**): 10 EGCG + 5 µg/mL CRO. (**C**): 10 EGCG + 8 µg/mL CRO. (**D**): 10 EGCG + 10 µg/mL CRO. CFU: colony-forming units. Control: MHB alone. The remaining groups were MHB containing the corresponding concentration of compounds. The results of the comparison of the EGCG in combination with CRO versus CRO used alone are shown below. *n* = 4 per group.

Overcoming antibiotic resistance is a paramount challenge in treating MRSA infections [43]. Given the demonstrated efficacy of EGCG in targeting key pathogenic pathways, we hypothesized that it might also impair bacterial resistance mechanisms. Transcriptomic analysis validated by qRT-PCR confirmed that EGCG significantly downregulated genes encoding penicillin-binding proteins (*pbp3*, *pbp4*) and peptidoglycan biosynthesis enzymes (*murA*, *murF*) (Figure 5). The directional concordance between techniques, despite variations in fold-change magnitude, supports the conclusion that EGCG may reduce MRSA’s resistance to β-lactam antibiotics. Therefore, the checkerboard assay was employed to investigate the synergistic effects of EGCG in combination with β-lactam antibiotics. The results showed that EGCG had a synergistic effect with cefoxitin, ceftriaxone, and ampicillin (FICI < 0.5) and had an additive effect with penicillin G (0.5 ≤ FICI < 1) (Table S4). Among them, the antibiotic with the strongest synergistic effect of EGCG was ceftriaxone (CRO). Thus, the MW2 strain was treated with serial concentrations of CRO in the presence or absence of 10 μg/mL EGCG. The results indicated that under combined treatment, increasing the concentration of CRO enhanced the synergistic antibacterial effect of EGCG and CRO and prolonged its duration of effectiveness (Figure 6). At the 2 h time point, the inhibitory effect of the combination of 10 μg/mL EGCG and 3 μg/mL CRO was comparable to that of 10 μg/mL CRO alone and maintained superior inhibitory activity until 6 h, while its overall effect was superior to that of the 20 μg/mL CRO group within 8 h (Figure 6A). At 2 h, the combination of 10 μg/mL EGCG with higher concentrations of CRO also showed similar efficacy to 10 μg/mL CRO alone but gradually exhibited stronger advantages over time. At 6 h, the antibacterial effects of the combination groups with 10 μg/mL EGCG and 5, 8, or 10 μg/mL CRO were all superior to that of the 20 μg/mL CRO group (Figure 6B–D). Furthermore, bacterial density in the combination group began to rebound after 4 h at 3 μg/mL CRO; this regrowth was delayed until 6 h and longer when CRO concentration was increased to 5 or 8 μg/mL, and was completely suppressed with no significant rebound observed at 10 μg/mL CRO after 6 h. These results fully demonstrated that the combination of EGCG and CRO produced a significant synergistic antibacterial effect and significantly reduced the required dosage of CRO while achieving comparable antibacterial efficacy.

## 4. Discussion

The rapid emergence of multidrug-resistant bacteria, particularly methicillin-resistant *Staphylococcus aureus* (MRSA), represents a critical threat to public health and underscores the urgent need for novel therapeutic strategies [2,5,6], highlighting the need for new strategies to combat *S. aureus* infections. Plant-derived natural products offer a promising source for drug development due to their multi-target potential and favorable safety profiles [44]. Among them, the natural polyphenol EGCG has attracted attention for its notable antibacterial and anti-inflammatory activities against MRSA [28]. In this study, EGCG exhibited broad-spectrum activity against MRSA, demonstrating concentration-dependent growth inhibition with an MIC_90_ of 10 μg/mL. This growth inhibitory effect may be closely related to its interference with pyrimidine metabolism. Pyrimidine metabolism results in the formation of pyrimidine nucleotides (namely uracil, cytosine and thymine) [45]. These are utilized for nucleic acid synthesis, energy production (UTP or CTP), and other essential cellular functions [46]. We observed significant downregulation of the de novo pyrimidine biosynthesis pathway genes: *pyrB* (aspartate carbamoyltransferase), *pyrC* (dihydroorotase), *pyrE* (orotate phosphoribosyltransferase), *pyrF* (orotidine-5′-phosphate decarboxylase), and *carA* (carbamoyl-phosphate synthase small subunit). Critically, the suppression of aspartate carbamoyltransferase, which catalyzes the first rate-limiting step in pyrimidine synthesis, directly impaired the conversion of glutamine and phosphopentose, reduced DNA and RNA synthesis, and consequently inhibited MRSA proliferation [47].

CWAs are important virulence factors of *S. aureus* and play a role in its pathogenic process [18]. Studies have shown that targeting the L-Lectin module in SraP, a CWA, can effectively inhibit host infection of MRSA. As a ligand-binding region of SraP, the L-Lectin module mediates the adhesion of *S. aureus* through the specific binding of key amino acids (e.g., Try367) to the sialylated receptor Neu5Ac on the surface of the host cell membrane. In this study, EGCG exhibited higher binding affinity to the L-lectin domain than its natural ligand, Neu5Ac, suggesting that EGCG might effectively block this adhesion pathway through competitive binding [21]. Consistent with this, EGCG showed a stronger inhibitory effect on cell adhesion and biofilm formation in MRSA WT strains than in ΔL-Lectin strains, confirming this domain as a key target for its anti-infection effect. Notably, the comparable growth inhibition of both strains suggests a L-Lectin-independent bacteriostatic mechanism, indicating that EGCG’s anti-infective action extends beyond direct killing to include adhesion and biofilm interference.

Considering the complexity of biofilm formation and systemic infection, we speculated that EGCG might have a broader virulence regulation network. ClfB binds to fibrinogen α-chain repeats5, human keratin-10, and loricrin to mediate MRSA colonization in the nasal cavity and skin [48,49]. We found EGCG inhibited the expression of *clfB*, thereby reducing the colonization rate of MRSA and the chance of MRSA infection in the host. Staphylococcal Protein A (SpA), an anti-phagocytic protein, promotes MRSA immune escape by non-specific binding to immunoglobulin IgG. EGCG promoted the reduction in *spa* expression level, which encodes SpA, aiding macrophages in capturing MRSA and reducing MRSA-induced hypersensitivity reactions and platelet damage [50,51]. Furthermore, the *lukS-PV* gene, which encodes a subunit of the *S. aureus* leukocidal Panton-Valentine leukocidin (PVL) that kills polymorphonuclear leukocytes (PMNs), particularly neutrophils, by perforating host cells, was significantly down-regulated following EGCG intervention [52]. Therefore, EGCG may reduce tissue damage and affect the immune response level by inhibiting the expression level of *S. aureus* exotoxin, which needs to be further verified by in vitro and in vivo experiments. It is unclear how EGCG regulates the expression of these genes. The two-component signal transduction system, ArlRS, is a global regulator of *S. aureus* virulence and regulates extracellular proteolytic activity, bacterial autolysis, capsule formation, and virulence factor production [53]. EGCG inhibited the expression of *arlR*—the gene encoding the response regulator of this system—suggesting a potential mechanism through which EGCG may interfere with virulence regulation in *S. aureus*.

Biofilms formed by *S. aureus* significantly enhance bacterial antibiotic resistance and are closely associated with severe infections [54,55]. In this study, EGCG had an inhibitory effect on the formation of MRSA biofilm. This effect may be achieved through multiple synergistic pathways. On the one hand, EGCG affected the expression level of MRSA CWAs, which play an important role in the early stages of biofilm formation [56]. Specifically, EGCG downregulated the expression of CWA genes including *sraP* and *clfB*, with the latter encoding a key biofilm mediator under calcium-limiting conditions [57], and concurrently upregulating *agrB*, a component of the Agr system that suppresses CWA expression [58]. These coordinated changes likely reduce surface protein levels, thereby impairing bacterial attachment during early biofilm formation. Additionally, EGCG-mediated downregulation of the spa gene may further compromise biofilm development, given the reported role of SpA in *S. aureus* biofilm-mediated infections [59]. On the other hand, EGCG also targets the pyrimidine metabolism pathway, which is crucial for biofilm integrity. Biosynthesis of pyrimidine nucleotides can affect biofilms by affecting the expression of the csgDEFG gene [42]. EGCG inhibited the de novo synthesis-related gene expression of the pyrimidine metabolic pathway, leading to impaired pyrimidine nucleotide production. This inhibition may further disrupt the synthesis of modified nucleotides—such as c-di-GMP—which function as key signaling molecules in biofilm formation [60]. Furthermore, inhibition of pyrimidine nucleotide biosynthesis can also lead to inhibition of eDNA synthesis, leading to destruction of biofilms [61]. Thus, EGCG may play its role in inhibiting the formation of MRSA biofilms by synergistically regulating bacterial adhesion, quorum sensing, and metabolic pathways.

In recent years, with the increase in infection with drug-resistant pathogens, combination drugs have become a new treatment idea [62]. Combination medications can reduce the concentration required for each drug, reducing the risk of drug toxicity and healthcare costs. At the same time, lower concentrations of antibiotics are less likely to induce resistance mutations in sensitive bacteria [63,64]. We found EGCG effectively reduced the expression of genes related to MRSA, β-lactam resistance, and peptidoglycan biosynthesis. β-lactam antibiotics mainly inhibit the synthesis of cell wall mucopeptide synthase, that is, penicillin binding proteins (PBPs), thereby hindering the synthesis of cell wall mucopeptides, causing bacterial cell wall defects and bacterial body expansion and lysis [65]. Peptidoglycan biosynthesis is a key step in bacterial cell wall synthesis; proteins such as MurA, MurF, and PBP2a are involved in the biosynthesis process of peptidoglycans [66]; and the gene expression encoding these proteins is significantly inhibited by EGCG, so it is speculated that EGCG may have a synergistic effect with β-lactam antibiotics, which was verified by the checkerboard method. EGCG and CRO exhibited a strong synergistic effect, with an FICI of 0.1875. As a common antibiotic in clinical practice, CRO is commonly used to treat systemic and local infections in humans and animals, such as peritonitis, sepsis, etc., but MRSA strains have developed resistance to it. Our results suggested that EGCG significantly improved the efficacy of CRO, and prolonged the duration of antibiotic action while reducing the amount of antibiotics. The combination of 10 μg/mL EGCG with 5 μg/mL CRO demonstrated enhanced efficacy compared to 20 μg/mL CRO alone after 6 h of treatment. This synergistic effect was maintained at 8 h, indicating that combining with EGCG could reduce the consumption of CRO by more than 75% while achieving comparable or superior outcomes.

In summary, our findings not only reveal the molecular mechanism of EGCG as a multi-potent antimicrobial, but provide a theoretical basis for the development of plant polyphenol-based MRSA combination therapy, especially for clinical intervention strategies for refractory biofilm infections and drug-resistant strains. However, this study still has certain limitations. First, the present findings are based entirely on in vitro systems. The therapeutic potential of EGCG alone and in combination with ceftriaxone, along with its correlation to clinically achievable tissue concentrations, requires future validation in animal infection models. A key translational hurdle is the poor bioavailability and low stability of EGCG in vivo. To overcome this, advanced delivery strategies such as nano-encapsulation could be employed to enhance its therapeutic potential [29,67]. Future work should also address the potential heterogeneity in responses across diverse clinical MRSA strains and extend the synergy analysis to other cornerstone anti-MRSA agents (e.g., vancomycin, linezolid, or daptomycin) to fully assess its broad utility as an adjunctive therapy [68]. Methodologically, future qRT-PCR analyses would benefit from employing multiple validated reference genes per MIQE guidelines to achieve more precise transcriptional quantification [69]. Taken together, despite the aforementioned limitations, the molecular insights and in vitro efficacy demonstrated here offer a valuable foundation for considering EGCG in the future development of adjunctive therapies against challenging MRSA infections.

## 5. Conclusions

The natural bioactive compound EGCG combats MRSA through a convergent, multi-target mechanism. Its anti-infective activity involves the direct binding to the L-lectin module of SraP to inhibit bacterial adhesion, coupled with the downregulation of key biofilm and virulence genes that are functionally linked to adhesion. Concurrently, EGCG disrupts pyrimidine metabolism to impair bacterial growth and suppresses β-lactam resistance genes, thereby resensitizing MRSA to antibiotics such as ceftriaxone. This coordinated attack on adhesion, metabolism, and resistance highlights EGCG’s potential as an adjunctive therapeutic agent against challenging MRSA infections.

## Data Availability

The data are available from the corresponding author upon reasonable request.

## Supplementary Materials

The following supporting information can be downloaded at: https://www.mdpi.com/article/10.3390/pathogens15010090/s1, Figure S1: Comparison of growth inhibitory rates between the WT group and the ΔL-Lectin group; Figure S2: Expression levels of genes associated with pyrimidine metabolism, virulence, and biofilm formation were analyzed by RNA-seq; Table S1: MIC detection of 12 strains used in this study; Table S2: Primers used in the study for construction of ΔL-lectin mutant; Table S3: Primers used in the study for qRT-PCR; Table S4: Synergistic antibacterial effects of EGCG combined with beta-lactam antibiotics on MW2.
