# Supplementary material for: Multitarget Mechanisms of (‒)‒Epigallocatechin-3-Gallate Against MRSA: From SraP L-Lectin Targeting to Synergistic Antibiotic Effects
Source: Pathogens. 2026 Jan 13;15(1):90. doi: 10.3390/pathogens15010090 (PMC12844705; doi:10.3390/pathogens15010090)
Supplement: Supplementary file 1 [file pathogens-15-00090-s001.zip › Supplementary material.pdf]

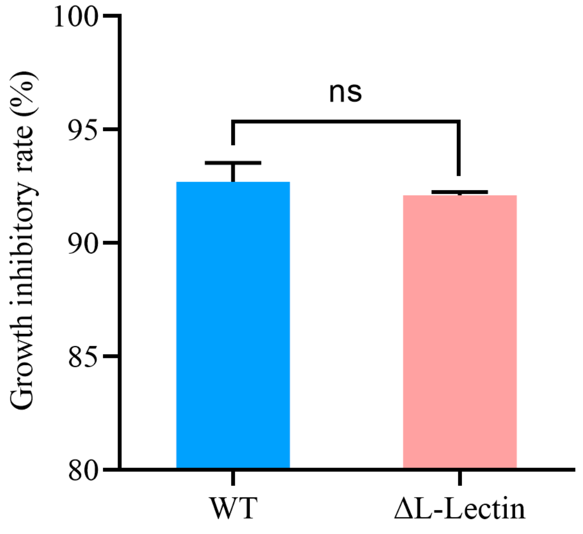

Figure S1. Comparison of growth inhibitory rates between the WT group and the ΔL-Lectin group. WT: 10 μg/mL EGCG-treated MW2 strains. ΔL-Lectin: 10 μg/mL EGCG-treated ΔL-Lectin strains. ns: non-significance.

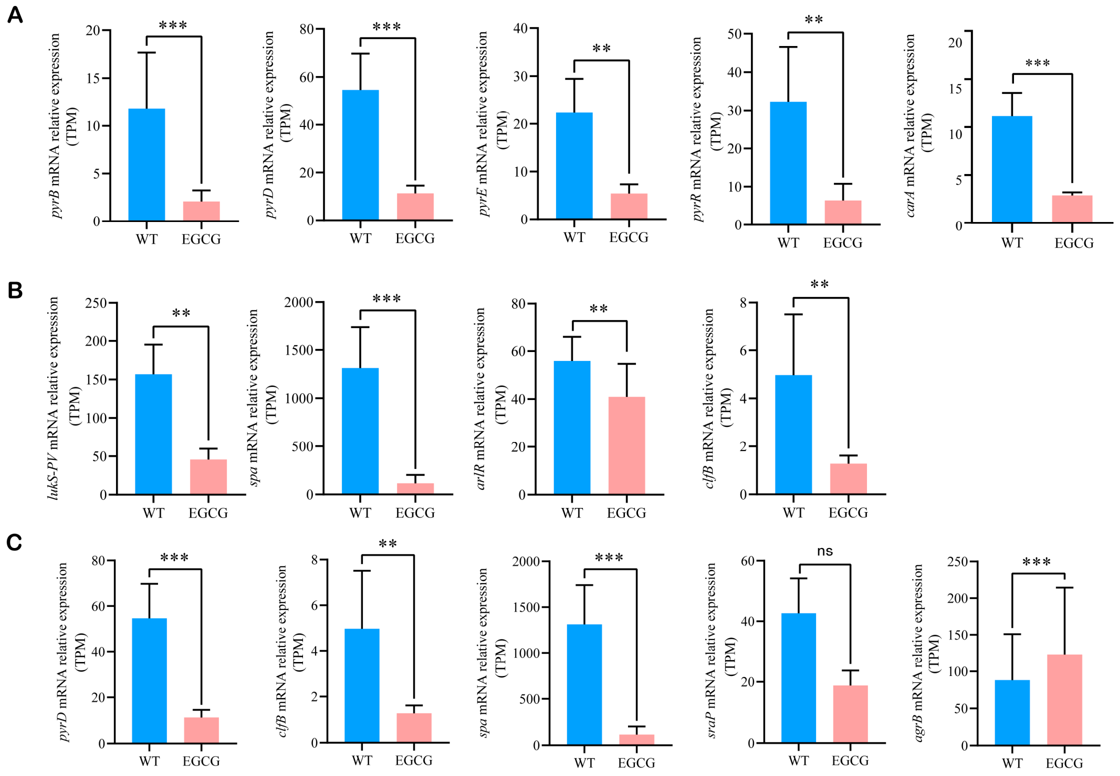

Figure S2. Expression levels of genes associated with pyrimidine metabolism, virulence, and biofilm formation were analyzed by RNA-seq. TPM: transcripts per million. WT: PBS-treated MW2 strains. EGCG: 10 μg/mL EGCG-treated MW2 strains. **$P < 0.01$, *** $P < 0.001$, ns: non-significance.

Table S1 MIC detection of 12 strains used in this study.

Strain number	Levofloxacin	Tetracycline	Gentamicin	Rifampicin	Chloramphenicol	Sulfamethoxazole	Cefoxidine	Ampicillin	Ceftriaxone	Penicillin G
92	8	64	32	4	≥ 512	32	4	256	4	256
9	256	256	32	128	256	512	256	512	≥ 512	32
39	128	256	128	≤ 1	≥ 512	≥ 512	64	512	64	64
22	256	256	128	8	≥ 512	8	4	256	8	16
20	16	32	16	8	≥ 512	≥ 512	64	128	128	16
203	64	16	32	2	≥ 512	≥ 512	8	256	16	64
64	128	256	64	≤ 1	≥ 512	≥ 512	16	256	16	32
50	64	256	64	≤ 1	≥ 512	≥ 512	16	128	64	64
180	16	128	4	≤ 1	≥ 512	≥ 512	16	128	64	4
51	128	128	8	≤ 1	512	16	16	128	128	8
128	256	128	256	≤ 1	≥ 512	≥ 512	16	256	128	16
MW2	2	128	4	≤ 1	≥ 512	4	64	256	256	8

Table S2 Primers used in the study for construction of ΔL-lectin mutant.

Primer name	Forward Primer (F)/ Reverse Primer (R)	Primer sequence（5'-3'）
L-lectin-up	F	AGGTGGATGGTATAGATATATTA
	R	TTGTGTAACAGCAGACTCTGTTGACATAGCTAAGCGACTG
L-lectin-down	F	CGCTTAGCTATGTCAACAGAGTCTGCTGTTACACAAGTGA
	R	TTGATACACTATTACTTTCAGAT
L-lectin-pKOR	F	TGAGCCTCGGAACCGGTACCATTCACGGTAAATATGTTGCA
	R	GGCAGTGAGCGCAACGCAATTTGATACACTATTACTTTCAGA
pKOR1-L-lectin	F	GTAATAGTGTATCAAATTGCGTTGCGCTCACTGCCCGCTT
	R	ATATTTACCGTGAATGGTACCGGTTCCGAGGCTCAACGTC
L-lectin-JD	F	AACAGCCGATTCCACAAGTGTAA
	R	TGAATTCGTTGCACTATCGTAAC
L-lectin-ter	F	TTTGCGTCAGCAGCGACGACA
	R	GTAAATTTGTCGCGCCACCTG

Table S3 Primers used in the study for qRT-PCR.

Gene name	Forward Primer (F)/ Reverse Primer (R)	Primer sequence（5’-3’）
pyrB	F	TCACGTGTCGCACGTAGTAA
	R	TTCTGCAAGCCCATGCCTTT
pyrD	F	ACACCTTGGATTCGGTGCTT
	R	ACGCGCTTCATAAGGTGTCA
pyrE	F	TGCAACAGCTGGTATTCCACA
	R	AGGCTTCAACTGCTGTGACT
pyrR	F	TGCCGCAATACAACGTACAG
	R	CCGTTCGACCAGTATACAGCA
carA	F	TTCCAGGGATTGCAGGTGTT
	R	AACGCCATCTGGAGCCATTG
lukS-PV	F	AATAACGTATGGCAGAAATATGGATGT
	R	CAAATGCGTTGTGTATTCTAGATCCT
spa	F	TCAGCTTTCGGTGCTTGAGATTCGT
	R	GCGCAACGTAACGGCTTCATTC
arlR	F	TATCCTTTTGTGGCTGACGA
	R	TGGGCTTGATTACGGTGC
clfB	F	CCTGATTTAGGTGCCTTTGC
	R	ACGAATGGCGATGTTGTAGC

Table S4. Synergistic antibacterial effects of EGCG combined with beta-lactam antibiotics on MW2

Antibiotic	$MIC_{antibiotic}$	$MIC_{combine}$	$FIC_{antibiotic}$	MIC_{EGCG}	$MIC_{combine}$	FIC_{EGCG}	FICI
Penicillin G	8	4	0.5	256	32	0.125	0.625
Cefoxidine	32	8	0.25	256	64	0.25	0.5
Ceftriaxone	256	16	0.0625	256	32	0.125	0.1875
Ampicillin	256	32	0.125	256	32	0.125	0.25

MIC: Minimum Inhibitory Concentration. FIC: Fractional Inhibitory Concentration.

FICI: Fractional Inhibitory Concentration Index.
